# Shade Tree Diversity, Cocoa Pest Damage, Yield Compensating Inputs and Farmers' Net Returns in West Africa

**DOI:** 10.1371/journal.pone.0056115

**Published:** 2013-03-08

**Authors:** Hervé Bertin Daghela Bisseleua, Daniel Fotio, Yede, Alain Didier Missoup, Stefan Vidal

**Affiliations:** 1 MDG Centre West and Central Africa, Dakar, Senegal; 2 Laboratory of Entomology, IRAD, Yaoundé, Cameroon; 3 Georg-August-University Goettingen, Department of Crop Science, Entomological Section, Goettingen, Germany; 4 Centre for Environmental Research and Conservation, The Earth Institute of Columbia University, New York, New York, United States of America; University of Zurich, Switzerland

## Abstract

Cocoa agroforests can significantly support biodiversity, yet intensification of farming practices is degrading agroforestry habitats and compromising ecosystem services such as biological pest control. Effective conservation strategies depend on the type of relationship between agricultural matrix, biodiversity and ecosystem services, but to date the shape of this relationship is unknown. We linked shade index calculated from eight vegetation variables, with insect pests and beneficial insects (ants, wasps and spiders) in 20 cocoa agroforests differing in woody and herbaceous vegetation diversity. We measured herbivory and predatory rates, and quantified resulting increases in cocoa yield and net returns. We found that number of spider webs and wasp nests significantly decreased with increasing density of exotic shade tree species. Greater species richness of native shade tree species was associated with a higher number of wasp nests and spider webs while species richness of understory plants did not have a strong impact on these beneficial species. Species richness of ants, wasp nests and spider webs peaked at higher levels of plant species richness. The number of herbivore species (mirid bugs and cocoa pod borers) and the rate of herbivory on cocoa pods decreased with increasing shade index. Shade index was negatively related to yield, with yield significantly higher at shade and herb covers<50%. However, higher inputs in the cocoa farms do not necessarily result in a higher net return. In conclusion, our study shows the importance of a diverse shade canopy in reducing damage caused by cocoa pests. It also highlights the importance of conservation initiatives in tropical agroforestry landscapes.

## Introduction

Human economy grows at the competitive exclusion of nonhuman species. Ecological changes due to agricultural intensification are known to increase anthropogenic biodiversity loss [1], [2]. However, conservation biologists and economists increasingly acknowledge the need to incorporate environmentally sustainable cocoa production strategies into conservation strategies [3], [4]. Recently, cocoa agro-ecosystems have received substantial attention because of their social, economic and ecological importance [5], [6]. Cocoa is important for national macroeconomic balances and provides livelihoods to millions of people in developing and developed countries.

Shaded plantations facilitate dispersal of forest fauna between fragments. Plant and animal biodiversity found within shaded cocoa systems could augment ecosystem services like pest control, pollination, weed control, fungal disease limitation, and soil fertility [7], [8], [9]. However, increasing and widespread intensification of management practices, including removal of shade trees and frequent weeding, is resulting in different cocoa production systems ranging from forest-like environments to full-sun cocoa [10]. How these different cocoa habitats differ in their fauna and flora, and how this affects functionally important species groups and ecosystem functioning is largely unknown. However, species diversity of birds and insects has functional consequences and influences ecosystem processes and services such as natural pest control [11]. Additionally, the type of interactions among species in an agro-ecosystem and the sensitivity of each species to different types of environmental fluctuations predict the stability of that system.

A considerable number of ecologists have acknowledged the role of cocoa agroforests as a refuge for biodiversity, specifically for ants, spiders and wasps [12]. Several studies have emphasized the role of ants in biological control in cocoa plantations [13], [14], [15], or their influence on other predators in agro-ecosystems [16]. Moreover, as cocoa plantations get intensified (with the reduction or elimination of shade trees); it is likely that the response of ant diversity to unpredictable outbreaks may vary. However, the extent to which cocoa agroforests are managed, with respect to the shade tree cover, species richness of the shade trees and herbaceous vegetation, and whether they provide valuable habitat and improve ecosystem functioning has barely been investigated in the West African cocoa belt [17]. Studies from Mesoamerica and Southeast Asia abound [18], [19], but cannot simply be transferred to West Africa considering the differences in management, tree phenology and structure and composition of ground-living herbaceous plants [20], [21].

The multi-strata cocoa agroforest in Cameroon harbor both rare and common species of aesthetic and cultural interest, and maintain valuable ecosystem services that are ensured by high species diversity [21]. Such wildlife-friendly farming approaches enable coexistence of agricultural activity and biodiversity in the same landscapes. Intensification may alter species diversity of relevance for conservation and ecosystem functioning [22], [23], [24]. We therefore need to predict consequences of agricultural intensification (specifically the reduction or elimination of shade) in order to develop pro-poor agroforestry strategies and incentives to conservation-friendly, ecologically complex agroforestry systems in West Africa. In addition, we must also strengthen the ecological knowledge of farmers to improve the farmer's ability to manage his/her local landscape [25].

This study focuses specifically on pest infestation and input use in cocoa agroforests with the aim of improving our understanding of how diversified and complex shade, in addition to biodiversity conservation, can provide ecosystem services such as biological pest control. We tested the hypotheses that, (i) shade tree removal may alter pest control and, (ii) reduction of inputs would enable coexistence of agricultural activity and biodiversity in the same landscape. The article highlights the contribution of complex shade agroforests in reducing pest infestation and input use. We also discuss recommendations derived from a different approach in conservation management of both cultivated forests such as traditional cocoa agroforests and the wider landscape of southern Cameroon (many of which are also applicable in other cocoa regions).

## Materials and Methods

### Study sites

In Cameroon, cocoa was originally grown by smallholders under a structurally and floristically diverse canopy of shade trees that provided a habitat for a high diversity of flora and fauna [26], [27]. The typical production system involves clearing virgin forests to plant new trees, and later replacing old cocoa plantations with food crops [28], [29]. Our study took place in five major cocoa-growing regions (Ngomedzap, Bakoa, Obala, Talba and Kedia) in the Central Region of Cameroon between 2°35' N and 4 °15' N and 11°48' and 11°15' E. The mean annual temperature is about 25 °C with a relatively small thermal variation. The mean annual rainfall is about 1600 mm per year. The five regions differed in land-use management ranging from less extensive (Ngomedzap), intermediate (Bakoa and Obala) to more intensive (Talba and Kedia) cocoa agroforests. Landscape characteristics are summarized in Table 1.

**Table 1:** Landscape characteristics of the regions.

Region	Rainfall regime (mm)	Age of cocoa plantation (yrs)	Agricultural land	Forest land
Ngomedzap	>1900	>50 “rustic plantation”	20% cocoa fields	70% Pristine forest
			10% annual crop (cassava, plantain)	With Forest reserve
Bakoa	<1100	∼30	50% cocoa fields	20% secondary forest No reserve
			25% annual field crops (maize, yams, citrus)	
			5% Patchy pasture fields	
Obala	>1300	∼40	70% cocoa fields	5% secondary forest No forest reserve
			25% annual crop fields of mixed crops (homegardens: cassava, groundnuts, maize, tomatoes etc…), agroforestry trees (citrus, safou, avocado, etc…).	
Talba	∼1200	15–20	70% Cocoa fields	25% pristine forest No reserve
			5% annual field crops (banana, plantain)	
Kedia	∼1050	8–15	65% cocoa fields	5% secondary forest
			25% annual field crops (maize) 5% pasture lands	

Four cocoa plantations ranging from 1 to 3 ha, and located at least 500 m from one another were selected in every region while ensuring that the plantations were managed exclusively by their owners using production techniques common to small landholders in the region [10]. The selected plantations differed in shade intensity, shade density, weed intensity, weed density and cocoa density. In each chosen plantation we assessed floristic (forest tree and herb species) and insect diversity. No specific permits were required for the described field studies and locations/activities. We received permissions from the cocoa growers associations from the selected regions to conduct the field studies. The locations and field studies are privately-owned by cocoa growers but not protected in any way. The field studies and locations did not involve endangered or protected species.

### Vegetation survey

We collected data on the vegetation characteristics within four 20×30 m plots in each plantation. We recorded the number of shade trees species. Unknown trees were given a unique morphospecies number. We estimated canopy cover within a 30 m radius circle at 10 subpoints within the circle; the center and at approximately 15 m N, S, E and W of the center subpoint. To estimate canopy cover we took readings with a hand-held concave densiometer at each of the 10 subpoints. To estimate canopy structure (depth), at each of the 10 subpoints we recorded the height of the lowest and highest canopy vegetation immediately above the subpoint. We used a digital rangefinder to improve our estimates of canopy height. The differences in the highest and lowest vegetation heights were used to estimate canopy depth at the 10 subpoints within each circle. We also recorded all herb species in 15 quadrates of 2×1 m per plot. Scientific and vernacular names (the latter given by local stakeholders) were recorded. Species that could not be identified in the field were identified at the National Herbarium of Cameroon (Yaoundé).

To represent land-use intensity, we created a shade index based on eight variables: number of trees, number of tree species, tree density, number of herbs, number of herb species, average tree height, percent shade cover, and percent herb cover. The mean of each variable was divided by the highest value of the same variable recorded in the plantation. We then summed the resulting values for all variables in one plantation, and divided this by the number of variables (i.e. eight) to obtain a value between zero and one for each plantation, where zero would represent the least diverse and one the most diverse shade. In each plot rainfall was recorded per day. For the analyses we used the mean of the average monthly rainfall per plot during the study period.

### Insect pests and natural enemy surveys

In each plantation we selected 30 cocoa trees at least 15 m apart, which we monitored weekly for pests and predators over two cocoa growing seasons, from March to December [30]. In each tree we quantified the total number of pods damaged by the cocoa pod borer (CPB; *Conopomorpha cramerella* Snellen), the total number of CPB holes on cocoa pods and CPB larvae per cocoa pod, the black pod rot (BPR; *Phytophthora megakarya* Brasier & M.J. Griffin), and the fresh feeding lesions caused by mirid bug *Sahlbergella singularis* Hagl. as well as the number of adult mirids.

On the same 30 selected trees, we sampled active ants between 9 AM and 1 PM [2] and the numbers of spider webs and social wasp nests. We also used 10 plastic observation plates (10 cm diameter) equipped with baits of about 4 g, composed of pieces of tinned tuna fish, honey, and cookie crumbs, to sample ground-foraging ants on 5 of the selected cocoa trees and 5 forest trees. Two persons monitored all plates on a subplot by observing each baited plate for 1 minute. For each ant species appearing on the plate, 5–10 specimens were caught with forceps and preserved in 70% ethanol for later identification. Ant species that occurred as singletons were sampled immediately to avoid missing them.

### Household and village surveys

In each region, we also randomly selected and interviewed 200 farmers, including the farmers among whom we sampled the biological information. We investigated their economically motivated preference for shade tree removal in their cocoa agroforests. We also collected socio-economic data (age and size of cocoa production area, total cocoa yield and revenue from cocoa production, costs for agro-chemicals including herbicides, pesticides and fertilizers, and agricultural technologies). We examined the differences in cocoa landholding, agrochemical costs, alternative forest products, cocoa yields and annual net returns per hectare using univariate analysis of variance.

### Statistical analysis

We use our different surveys per agroforest to generate sample-based rarefaction curves (MaoTao estimates) with EstimateS Version 8.2.0 [31] to compare plant (tree and herb) richness. We rescaled samples based rarefaction curves to the number of individuals to best compare richness between regions [32], [33]. Using the Chao-Jaccard Estimated Abundance Indices [34], we re-computed Chao1 for abundance distribution with a coefficient of variation higher than 0.5 and calculated first order jack-knife estimators of species richness.

A multiple linear regression analysis was used to describe the relationship between predators (ant/spiders/wasps) and vegetation variables (number of trees, number of tree species, tree density, number of herbs, number of herb species, average tree height, percent shade cover, and percent herb cover). We also used simple linear regression analysis to describe the relationship between predators (ant/spiders/wasps) and the presence of native or exotic shade tree species as well as the relationship between pod rot and shade index. Because of the likeliness that environmental gradients such as rainfall and the shade cover gradient confound each other we conducted general linear model and correlation analyses controlling for rainfall against biodiversity data. We also conducted non-parametric tests with all biodiversity data (predator richness, tree and herb richness) and shade index to provide alternatives to ANOVA.

Data on species richness of ant and the number of spiders/wasps were analyzed by multiple regressions against shade index and rainfall. Where needed we additionally perform kruskal Wallis test on biodiversity data (predator richness, tree and herb richness) and shade index. General linear model and correlation analyses conducted in Systat 11 [35] were also used to analyze data on yields. We used yield as the dependent variable and shade index, predators (ant/spiders/wasps) as independent variables in the multivariate regression analysis to separate the influence of management strategy from confounding factors such as age. We also looked at the relationship between the input costs and the net return. We used log-transformed data on species count to meet the condition of normality.

## Results

### Species richness and similarity of plants

**Figure 1:**
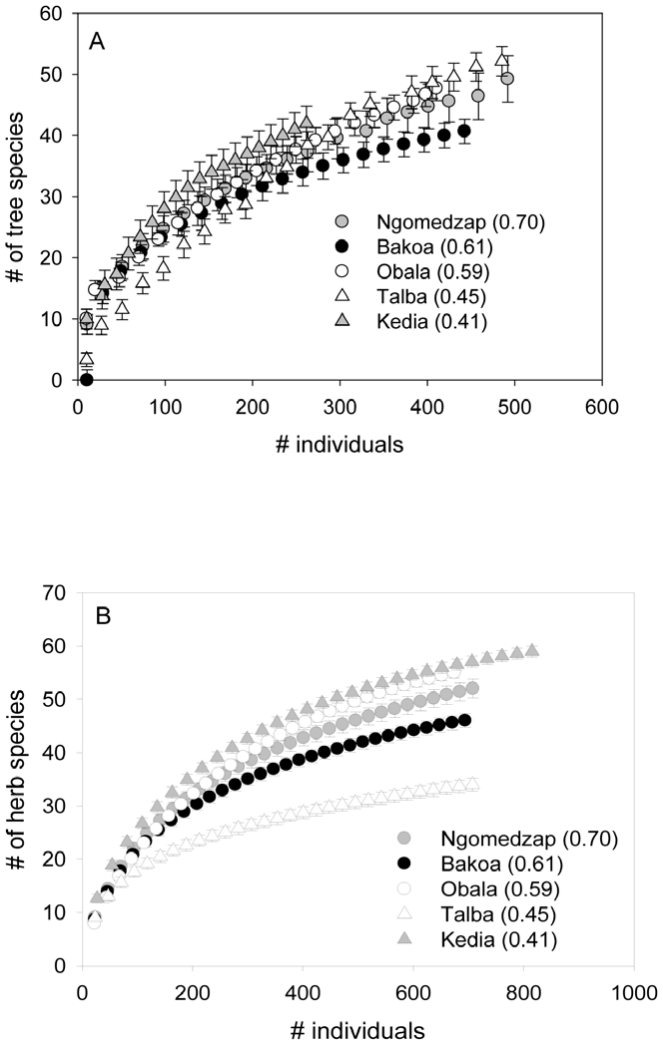
Species accumulation curves for trees (a) and herb species (b) in cocoa agroforests in relation to shade index. Error bars show 95% confidence intervals and non-overlapping bars show significant differences between shade indexes. Figures in parentheses are average values of the shade index for each region.

We recorded a total of 102 tree species and 260 herbaceous species belonging to 56 families of trees and 113 families of herbs, respectively. The shade index differed in each region with cocoa plantations near pristine forests (Ngomedzap) having the highest index, cocoa plantations in forest galleries (Bakoa) and in homegardens (Obala), having an intermediate index and cocoa plantations near secondary forests (Talba) and in artificial forests (Kedia) having the lowest shade index (Table 2). The uses for each tree species is detailed in Table 3. Species similarity between regions was low for trees and herbs. Of all tree and herb species recorded, 31% were shared between Bakoa and Ngomedzap, 27% were shared between Obala and Ngomedzap and only 16% were shared between Ngomedzap and Kedia; and between Talba and Ngomedzap. However, similarity of tree and herb species did not significantly differ between regions (ANOVA, Chao-Jaccard Estimated (F_4, 19_ = 0.23, P = 0.87) for tree species, Chao-Jaccard Estimated (F_4, 19_  = 2.8, P = 0.13) for herb species). The ANOVA of the shade indices revealed statistically significant differences among the five regions (F_4, 19_ = 10.94, P<0.001). Tree and herbaceous species richness significantly decreased with decreasing shade index (Tree species (F_1, 19_ = 14.7, P<0.0001, Fig. 1a); Herb species (F_1, 19_ = 10.3, P<0.0001, Fig. 1b).

**Table 2:** Variables used to calculate shade index in cocoa agroforests in Cameroon.

Variable name	Description	Minimum	Mean (SE)	Maximum
# Tree individuals	Number per hectare	17	88.9 (15.0)	220
# Tree species	Number of shade per hectare	4	8.0 (0.6)	13
Shade cover	In percent, measured above ground	25	73.3 (4.0)	95
Mean tree height	In meter, shade trees with dbh >5 cm	36	54.8 (2.4)	72.0
# Herb individuals	Number of herbs per hectare	72	103 (33.2)	216
# Herbaceous species	Number of herb species per hectare	12	25.1 (1.4)	36
Herbaceous cover	In percent, measured in quadrate	5	45.0 (7.5)	100
Cocoa tree density	Number per hectare	900	1230.5 (54.7)	2000

N.B. min and max were calculated over all 5 regions using all cocoa plantations.

**Table 3:** List of 43 common forest tree species recorded and used as explanatory variables to explain shade index in cocoa agroforests in Cameroon.

Species	Family	Local/common name	Conserva-tion star*	Economic importance/uses				
				Timber	Food/spice	Medicine	Fuel-wood	Other
*Spondias lutea* Linn.	Anacardiaceae	Cassimaga			X(fruit)			
*Xylopia aethiopica* (Dunal) A. Rich	Annonaceae	Akui		X	X(spice)	X		
*Alstonia boonei* De Wild.	Apocynaceae	Ekouk/Emien	Green			X		
*Voacanga africana*	- \\ -	Voacanga				X		
*Elaeis guineensis* Jacq.	Arecaceae		X		X(Oil)			Wine
*Newbouldia laevis* (P.Beauv.) Seem.	Bignoniaceae	Nouentchè/Mbikam				X		
*Spathodea campanulata* P. Beauv. Subsp.	- \\ -	Evovone/Tulipier	Green				X	
*Ceiba pentandra* Gaertn.	Bombacaceae	Doum/Fromager	Pink					Shade
*Cordia platythyrsa* Baker	Boraginaceae	Ebe/African cordia	Blue	X		X		
*Canarium schweinfurthii*Engl.	Burseraceae	Abel/Aiele	Red	X	X (fruit)			
*Dacryodes edulis* (G.Don) H.J. Lam	- \\ -	Plum/Safou	Green		X(fruit)			
*Monopetalanthus microphyllus*A. Chev.	Caesalpiniaceae	Ekop/Yellow ndoung		X				
*Musanga cecropioides*	Cecropiaceae	Asseng/Parasolier	X					
*Terminalia superba* Engl. & Diels	Combretaceae	Akom/Fraké	Pink	X				
*Diospyros* spp.	Ebenaceae	N′nom Elem		X				
*Discoglypremna caloneura* (Pax) Prain	Euphorbiaceae	Dambala						
*Hevea brasiliensis* Muell. Arg.	- \\ -							Latex
*Ricinodendron heudelotii* Mull. Arg.	- \\ -	Ezezang/Djansang	Green		X			
*Guibourtia demeusei*(Harms) J. Léonard	Fabaceae	Essingang/Bubinga		X				
*Pterocarpus soyauxii* Taub.	- \\ -	Mbel/Red Padauk	Red	X				
*Hypodaphnis zenkeri* Stapf.	Lauraceae	Ataag				X		
*Petersianthus macrocarpus* Liben	Lecythidaceae	Abing/Abale		X				
*Entandrophragma cylindricum* Sprague	Meliaceae	Assie/Sapelli	Red	X				
*Khaya senegalensis*	- \\ -	Mahogany		X				
*Lovoa trichilioides* Harms	- \\ -	Bibolo/Dibétou		X				
*Albizia adianthifolia* W.Wight	Mimosaceae	Sal'yeme/Bangbaye	Pink			X		
*A. ferruginea* (Guill. & Perr.) Benth.	- \\ -	Evouvous/Ossoto'o	Pink			X		
*A. zygia* (DC) J.F. Macbr.	- \\ -	Sal'yeme/Ketomb	Pink			X		
*Piptadeniastrum africanum* Brenan	- \\ -	Atui/Dabema	Red	X		X		
*Tetrapleura tetraptera* Taub.	- \\ -	Akpa				X		
*Ficus exasperata* Vahl.	Moraceae	Akol/Akole	X			X	X	
*Ficus mucuso* Welw. ex Ficalho	- \\ -	Toily/Figuier	X			X		
*Micicia excelsa* (Welw.) C.C. Berg.	- \\ -	Abang/Iroko	Scarlet	X				
*Morus mesozygia* Stapf.	- \\ -	Abang/Yellow iroko		X				
*Morinda lucida* Benth.	Rubiaceae	Akeng		X				
*Cola acuminata*	Sterculiaceae	Kola			X (fruit)			
*Cola nitida* (Vent.)Schott & Endl.	- \\ -	Kola			X (fruit)			
*Cola lepidota* K. Schum.	- \\ -	Kola	Gold		X (fruit)			
*Mansonia altissima* A. Chev/Chev.	- \\ -	Nkul/Bete	Gold	X				
*Triplochiton scleroxylon* K. Schum.	- \\ -	Ayous	X	X				
*Duboscia macrocarpa* Bocq.	Tiliaceae	Akak				X		
*Eribroma oblongum* (Mast) Pierre.	Ulmaceae	Eyong		X				

* In descending order of conservation importance: black, gold, blue, scarlet, red, pink and green [36].

*Source*: Household cocoa farmer survey and field survey.

### Species richness and similarity of natural enemies

**Figure 2:**
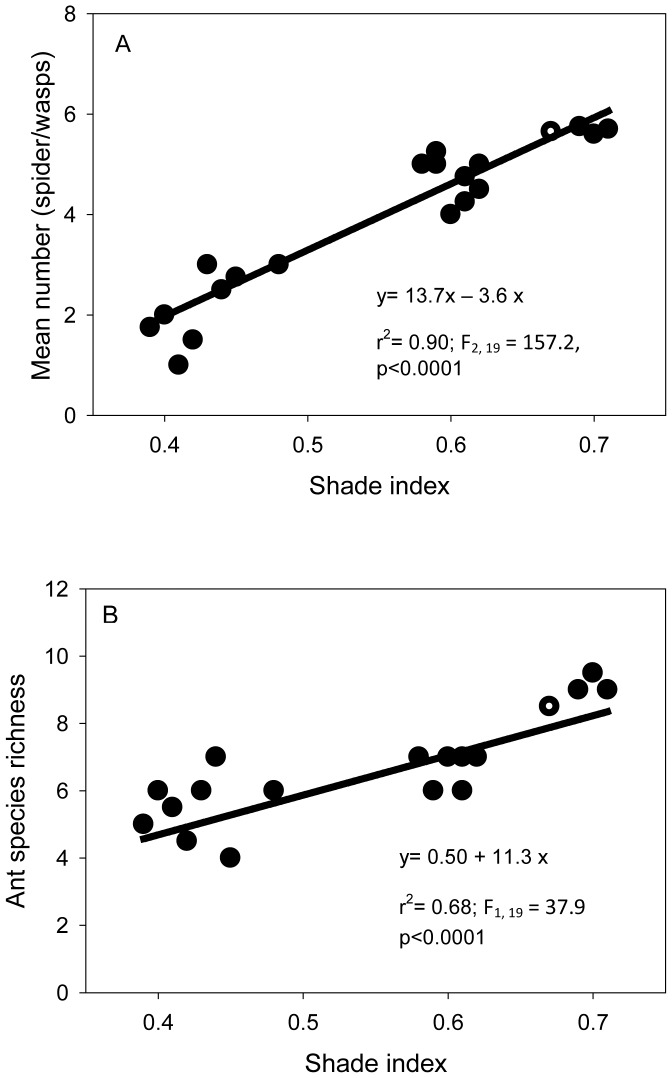
Mean number of spider webs/wasp nests (a), and ant species richness (b) in cocoa agroforests in relation to shade index.

We recorded 38 species of ants within the cocoa agroforests, which represent between 56% and 73% of the maximum number of species determined by commonly used estimators for species richness (Chao: 72.55±12.90; First order jacknife: 55.58±11.87). Species richness of ants significantly decreased (y = 0.50+11.3x, r^2^ = 0.68, F_1, 19_ = 37.9, P<0.0001) with decreasing shade index (Fig. 2b). Ant species similarity between cocoa agroforests was higher than that of trees and herbs. An average of 51% of ant species was shared between agroforests. Again, similarity of ant assemblages in cocoa agroforests did not differ significantly with cocoa plantation location (ANOVA, Chao-Jaccard Estimated (F_1, 19_ = 0.9, P = 0.52). The number of spider webs and wasp nests was significantly higher (F_2, 19_ = 157.2, P<0.0001) at higher shade indices (Fig. 2a). We also noted that species richness of ants significantly increased (F_1, 19_ = 38.0, P<0.0001) with increasing shade indices (Fig. 2b).

### Factors affecting species richness and pest damage

**Figure 3:**
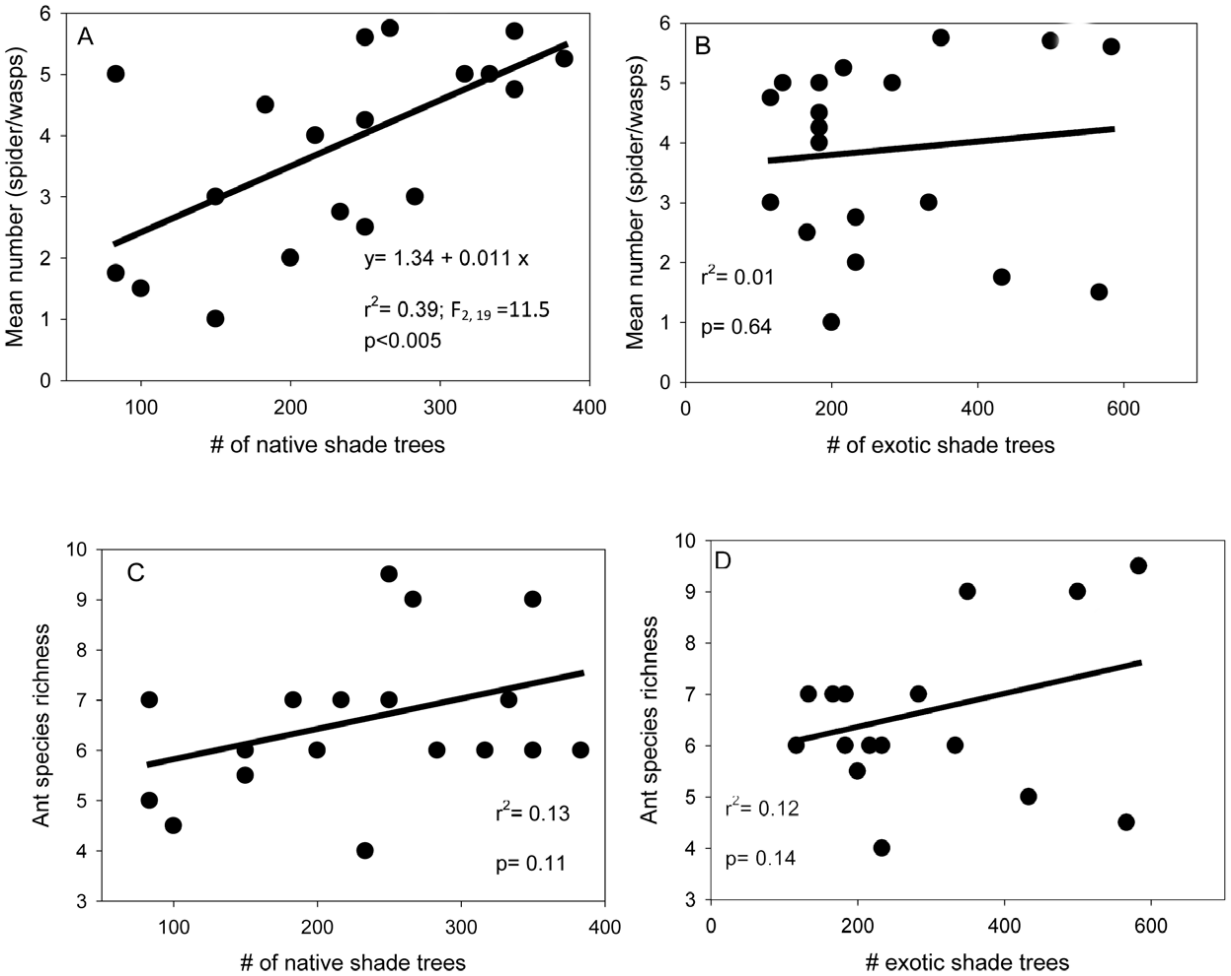
Relationship between the mean number of spider webs (*a* and *b*), ant species richness (*c* and *b*) and the type of shade trees (native and exotic) in cocoa agroforestry systems.

The number of spider webs and wasp nests significantly increased with increasing density of native shade trees (F_1, 19_ = 11.5, r^2^ = 0.39, P<0.005) (Fig. 3a). This number also tends to decrease with the density of exotic shade trees (Fig. 3b). Density of native and exotic shade trees did not have significant effects on ant richness (Fig. 3c and 3d). Rainfall and shade cover were correlated to some extend (Pearson's correlation, F_2, 19_ = 4.9, r^2^ = 0.22, P<0.05). However, ant richness was positively related with shade cover (F_1, 19_ = 12.2, r^2^ = 0.40, P<0.01), and herbaceous cover (F_1, 19_ = 12.7, r^2^ = 0.40, P<0.001). In the multivariate regression analyses, predator (ant richness and number of spider webs/wasps nests) richness was significantly affected by the percentage of shade cover, herb cover, and regions, respectively (F_1, 19_ = 14.8, P<0.0001, r^2^ = 0.70). Predator richness (ant richness and number of spider webs/wasps nests), the number of herbivores and the rate of herbivory were not affected by rainfall in all our analyses. The number of herbivores (mirid bugs and cocoa pod borers) and the rate of herbivory on cocoa decreased with increasing shade index (number: y = 26.9–36.9x, r^2^ = 0.76, F_1, 19_ = 57.8, P<0.0001; herbivory: *y* = 85.1–97.5*x*, *r^2^* = 0.64, F_1, 19_ = 32.4, P<0.0001). Pod rot caused by *Phytophthora megakarya* did not show any relationship with the shade index. The number of herbivores and the rate of herbivory showed a positive correlation with ant richness (number: F_1, 19_ = 36.8, r^2^ = 0.67, P<0.0001; rate of herbivory: F_1, 19_ = 22.6, r^2^ = 0.56, P<0.0001) and the number of spider webs and wasp nests (number: F_2, 19_ = 69.1, r^2^ = 0.78, P<0.0001; rate of herbivory: F_1, 19_ = 30.6, r^2^ = 0.63, P<0.0001).

**Figure 4:**
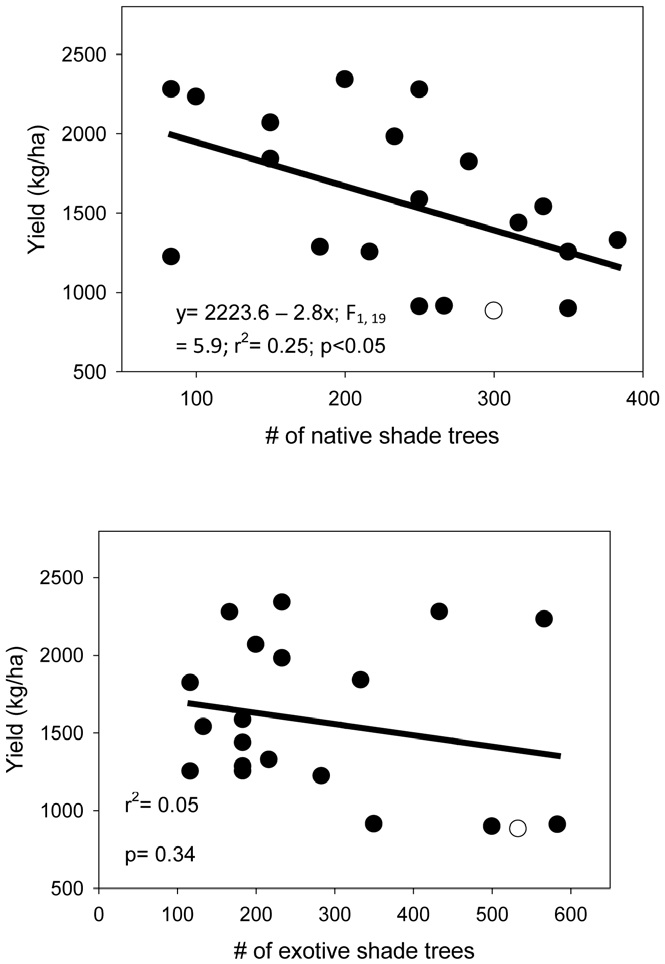
Yield-shade trees (native and exotic) relationship in cocoa agroforestry systems.

In the multivariate regression analyses, cocoa yield was significantly affected by the percentage of shade index, predator richness (ant, spider, wasps) and the age of cocoa trees (F_1, 19_ = 58.9, P<0.0001, r^2^ = 0.95). Native shade trees negatively affected yield (F_1, 19_ = 5.9, r^2^ = 0.25, P<0.05) as compared to exotic shade trees (Fig 4). Yield was significantly higher at shade and herb cover <50% (Shade cover: F_1, 19_ = 14.83, P<0.001; Herb cover: F_1, 19_ = 34.77, P<0.0001).

### Impact of shade index on annual return

When analyzing cocoa farmer survey we found that the management of shade trees significantly differed (F_4, 19_ = 78.2, P<0.0001) among regions. An average of 56% of farmers removed shade trees from their cocoa field. This figure included 4% of farmers in Ngomedzap (highest shade index), 58% in Bakoa, 69% in Obala and more than 72% of farmers in Talba and Kedia (lowest shade index). Reasons mentioned by farmers for shade removal were to reduce the incidence of pod rot and to increase yields. However, some shade trees were retained by farmers in their farms for fruits (70% of respondents), medicine (13%of respondents), timber (15% of respondents) and local spice (2% of respondents), such as the njangsang tree (*Ricinodendron heudelotii*) and the bush mango (*Irvingia gabonensis*).

**Figure 5:**
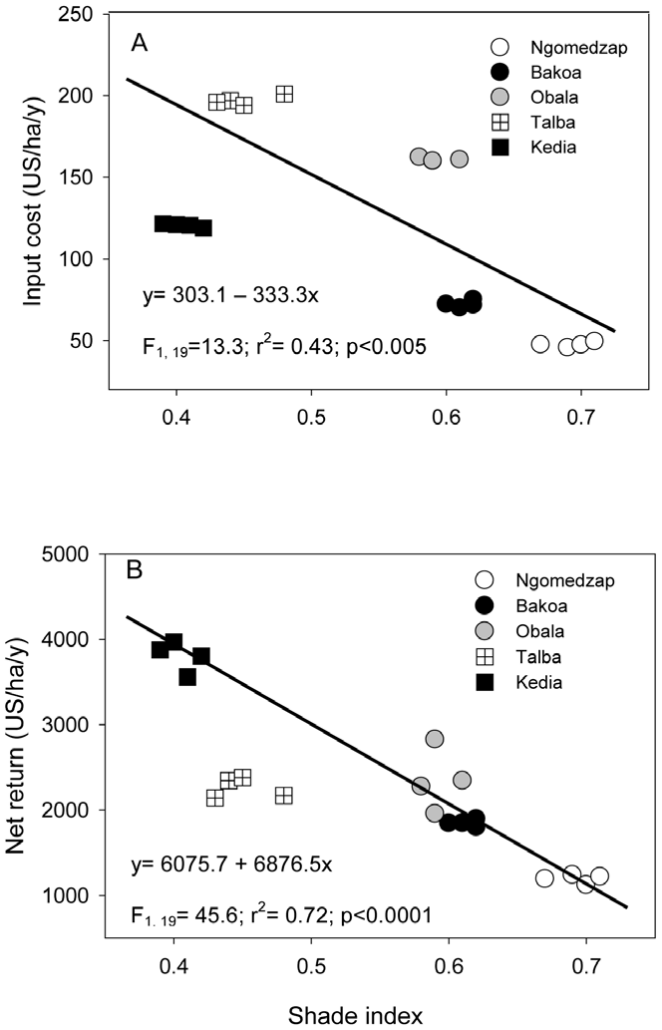
Cost of input (pesticides and labour) (a) and net returns (b) in cocoa agroforests in relation to shade index.

Farming household surveys from the 5 regions also revealed that intensified cocoa production increased annual net returns from US$ 1194/ha on plots with 0.69 shade index to US$ 2349/ha with 0.59, and to US$ 3801/ha on plots with 0.41 shade index (for a less diverse farm) (Fig. 5). However, we observed that higher inputs in the cocoa farms did not necessarily result in a higher net return (y = 1490.5+6.7x, r^2^ = 0.18, F_1, 19_ = 3.87, P = 0.06).

## Discussion

We linked a biodiversity estimate to a management indicator on cocoa agroforests, thereby covering the full range from extensive to extremely intensive land use pattern. When differences in environmental conditions had been accounted for, we found evidence that plant species richness declined with increasing land use intensity.

We found that shade cover and environmental gradient such as rainfall do not confound each other. From all analyses controlling for rainfall, we found that although there is a correlation between shade and rainfall (r = 0.47), both variable do not impact biodiversity data in a similar way. Rainfall in cocoa agroforests in southern Cameroon is not the predictor of diversity of predators (ant, spiders and wasps) and plants. Instead, the shade index per plot and the shade tree diversity were more suitable for predicting diversity of ants, spiders, and wasps, respectively. We also found that under same rainfall condition, shade management by farmers has a significant impact on predator and plant richness. Because shade is strongly correlated with all biodiversity data, we focused our analyses on shade impacts on biodiversity data and yields and we have downgraded rainfall effects. Plant species richness is often closely related to the diversity of other trophic levels [9]. We also found that land use changes are driven by well-known socio-economic factors and culturally mediated innovations [36]. These observations highlight synergies that emerge from diversified cocoa agroforests and the conditions necessary to move from an unsustainable syndrome of production to a sustainable one. To our knowledge, this is the first study examining the relationship between biodiversity, agricultural matrix and pest control in cocoa agroforests in tropical Africa.

Our results document that differences in management among regions, specifically shade and herb layer management between smallholders strongly impacts cocoa landscapes and ecosystem service, such as biological pest control. We observed that shading and choice of shade trees are separate variables in the management choices of the smallholders, and consequently, these factors are correlated only to some extent. Common management practices in cocoa agroforests tend to decrease tree diversity over time. These include the progressive thinning of shade canopies (partly motivated by the need to maximize yields; [2]) and official recommendations to substitute old forest trees by the often exotic faster growing leguminous species [36] in order to provide conditions for soil rejuvenation [37], [38]. The mix of exotic and native species may not produce enough resources, such as fruits and breeding sites, needed for beneficial insects. Thus, the high proportion of exotic species in cocoa agroforests may contribute to the relatively low maintenance of a forest-based beneficial fauna [19], [39]. For example, greater diversity of shade trees in cocoa plantations was positively related to ant and parasitoids richness, and thus supported more natural enemies [6], [40]. Shade reduction may also increase the spread of invasive species, such as ants, in cocoa agroforests [41].

Our data on herbivores and herbivory supported the hypothesis that density of functionally monophagous herbivores will be reduced with increasing shade index [11]. Farms with greater vegetation heterogeneity and thus greater functional diversity of ants, spider and wasp species could exhibit stronger resilience of services after climatic disturbances or outbreaks through “insurance” species [42], [1]. Moreover, our results showed a positive association between ant richness, wasp nests, spider webs and shade indices. It is known that in cocoa agroecosystems ants play important roles in biological control by chemically deterring pest feeding [13] or directly by preying upon them. A higher richness of ants may enhance their ability to adapt and respond to changing conditions such as pest outbreaks or exploit new resources efficiently. However, the increased ecosystem function is not only due to diversity *per se* but rather the intraspecific differences in foraging or behaviour within beneficial insect communities that help to enhance the response to herbivory or to boost functionality under the insurance hypothesis [43].

The extent and diversity of the herbaceous layer only moderately affected spider and wasp numbers as compared to shade cover and tree richness, which suggests that canopy structure rather than herbs are the key variable for most parasitoids species and predators, such as spiders, in cocoa agroforest landscapes. Vegetation heterogeneity has been highlighted several times as being a surrogate for habitat suitability for beneficial insects in human dominated landscapes [44], [4], but this is the first time these variables were addressed at the scale of contrasting land-use types.

Our results showed that the matrix quality is important in the relationship between insect pests such as the cocoa pod borers and natural enemy control by wasps. Diverse cocoa agroforests represent a good quality matrix that promotes migration among fragments and maintains populations as meta-populations and therefore maintains biodiversity and ecosystem services at the landscape level [45]. Less diverse cocoa systems represent a low quality matrix that would hinder migration of beneficial insects such as wasps [4]. The lack of migration thus may cause local (within fragment) extinctions to turn into regional extinctions. Consequently, the nature of the agroecosystems that make up that matrix is important, not only as a potential repository of biodiversity, but also as a habitat through which organisms can migrate from fragment to fragment (i.e. the matrix). Therefore, to optimize the attractiveness of cocoa agroforests to beneficial insect species, the nature of cocoa plantations as part of the landscape matrix should be considered in term of species composition of the planned and unplanned crop and noncrop biodiversity.

We showed for smallholder agroforests that higher inputs do not necessarily result in a higher net return. This finding is remarkable because it has identified win-win situations in biodiversity-yield relationship in species-rich agroforests. Conserving biodiversity in these systems is associated with maintaining a diversity of shade trees, rather than simply the number of trees per se, combined with moderate inputs of pesticides and labor per unit area that will enhance biological pest control [6]. This suggests the possibility of establishing premium prices to promote shade tree diversity and habitat complexity in tropical human-dominated landscapes with the purpose of conserving biodiversity. Therefore, conservation of highly sensitive taxa should take into account lower yields resulting from diverse shade. Furthermore, analyses of the relationship between yield, shade index and net return suggest that increasing premium values may generate a dramatic shift from a plantation with high yield but low species richness to a plantation with low yield and high species richness. Nevertheless, high yields realized by intensification do not necessarily reduce functional biodiversity if a proper shade-vegetation structure is maintained. Policies and incentives aiming at helping cocoa farmers to overcome the costs of conversion from low-biodiversity systems to more diverse systems may, therefore, generate simultaneous increases in biodiversity and net income. Conservation programs of traditional land-use strategies must encourage cultural preferences for shade tree diversity and habitat complexity of tropical dominated-human landscape. Additionally, education of smallholders about unacknowledged ecosystem services provided by diversified and heterogeneous shade systems could further promote the implementation of certifications schemes. Such incentives will enhance the conservation value of traditional cocoa agroforests as an important refuge for tropical biodiversity and sources of valuable ecosystem services.

## Conclusion

The results of this study provide a conceptual framework for conservation initiatives in cocoa agroforest landscapes. Initiatives could be most (cost-) effective if they are preferentially implemented in low-intensity cocoa agroforestry systems that still support high levels of biodiversity. Our models show no simple trade-off between biodiversity and net income. However, a threshold in species richness at a 0.5 shade index in cocoa agroforests that is economically and ecologically profitable should be encouraged to balance economic and ecological needs.

This applies not only at the national level, but also at the international level, and highlights the importance of conservation initiatives on tropical human-dominated landscape of West Africa that host some of the most species rich farmlands, but are severely threatened by intensification [21]. Incentives from payment-for-ecosystem services and certification schemes should encourage farmers to keep heterogeneous shade tree cover. Conservationists and policy makers should nevertheless be aware that measures required to effectively conserving biodiversity and targeted species in these landscapes need to more drastically reduce land use intensity and will therefore be more costly. Participatory knowledge sharing between farmers, agronomists and ecologists will help to encourage heterogeneous shade systems that balance economic and ecological needs and provides a ‘diversified food-and-cash crop’ livelihood strategy.
