# Correction: Shade Tree Diversity, Cocoa Pest Damage, Yield Compensating Inputs and Farmers' Net Returns in West Africa

**DOI:** 10.1371/annotation/ce51ca58-84aa-4ba4-be8c-86cb46d35ed1

**Published:** 2013-06-18

**Authors:** Hervé Bertin Bisseleua Daghela, Daniel Fotio, Yede, Alain Didier Missoup, Stefan Vidal

The first author's name was spelled incorrectly. The correct name is: Hervé Bertin Bisseleua Daghela. The correct citation is: Bisseleua Daghela HB, Fotio D, Yede, Missoup AD, Vidal S (2013) Shade Tree Diversity, Cocoa Pest Damage, Yield Compensating Inputs and Farmers' Net Returns in West Africa. PLoS ONE 8(3): e56115. doi:10.1371/journal.pone.0056115. The correct abbreviation in Author Contributions is: HBBD.

