# Correction: Shade Tree Diversity, Cocoa Pest Damage, Yield Compensating Inputs and Farmers' Net Returns in West Africa

**DOI:** 10.1371/journal.pone.0101901

**Published:** 2014-07-14

**Authors:** The PLOS ONE Staff

In the second sentence of the “Insect pests and natural enemy surveys” section, the cocoa pod borer was incorrectly named *Conopomorpha cramerella*. The correct name is *Characoma stictigrapta*.
